# A Transformer for Reaction-Aware Compound Explorations with GFlowNet in QSAR-Guided Molecular Design

**DOI:** 10.1021/acs.jcim.6c00181

**Published:** 2026-04-25

**Authors:** Shogo Nakamura, Nobuaki Yasuo, Masakazu Sekijima

**Affiliations:** † Department of Life Science and Technology, 98406 Institute of Science Tokyo, Midori-ku, Yokohama 226-8501, Japan; ‡ Tokyo Tech Academy for Convergence of Materials and Informatics (TAC-MI), Institute of Science Tokyo, Meguro-ku, Tokyo 152-8550, Japan; § Department of Computing, 542793 Institute of Science Tokyo, Midori-ku, Yokohama 226-8501, Japan

## Abstract

In drug discovery tasks, achieving a balance between high biological activity toward therapeutic targets and synthetic chemical feasibility is critically important. While the recently proposed deep learning-based molecular generation models have enabled explorations of vast chemical spaces, most existing approaches do not consider synthetic routes for generated compounds. To address this issue, TRACE-GFN is proposed for molecular optimization; this method incorporates chemical reaction pathways into a quantitative structure–activity relationship (QSAR)-guided molecular design procedure. The method integrates a transformer model to explicitly learn chemical reactions with a generative flow network (GFlowNet) that efficiently samples diverse candidates. In benchmark experiments involving dopamine receptor D2 (DRD2), AKT serine/threonine kinase 1 (AKT1), and C-X-C motif chemokine receptor 4 (CXCR4), TRACE-GFN demonstrated the ability to identify compounds with high QSAR values while maintaining strong diversity, outperforming the existing molecular generation models. These results demonstrate that the proposed model can efficiently explore promising compounds while accounting for real-world chemical reactions. The source code is publicly available under an MIT license at https://github.com/sekijima-lab/TRACE-GFN.

## Graphical abstract

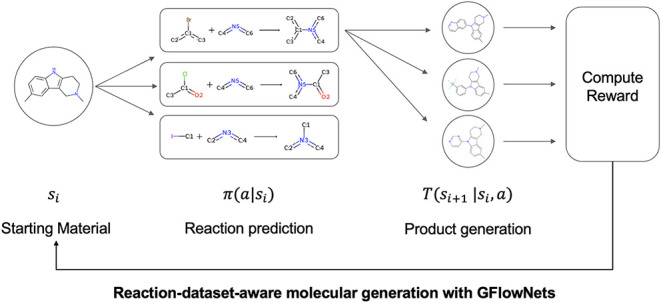

## Introduction

The design and organic synthesis of molecules that are capable of interacting with target proteins remain crucial challenges in the drug discovery field. However, the development of drugs has become increasingly difficult, and some drugs take more than 12 years to enter the market and cost an average of $2.6 billion.^1,2^ In recent years, modalities have diversified to include RNA, targeted protein degraders, cyclic peptides, and antibody–drug conjugates (ADCs).^3^ Despite these advances, small molecules remain important because of their high cell membrane permeability, oral bioavailability, and scalable manufacturing characteristics.^4^ Traditionally, high-throughput screening (HTS) and virtual screening (VS) techniques such as molecular docking have been employed for small-molecule design.^5,6^ More recently, deep learning-based molecular generation has emerged as a rapidly advancing approach, enabling explorations of much broader chemical spaces without compound libraries.^7^

Molecular generative models have been developed using diverse chemical representation methods, including the simplified molecular input line entry system (SMILES),^8^ molecular graphs, and three-dimensional molecular structures.^9^ These models utilize deep learning architectures such as recurrent neural networks (RNNs),^10–13^ variational autoencoders (VAEs),^14,15^ transformer models,^16–18^ and diffusion models.^19–21^ Recent advances have also included methods for generating molecules with specified interaction patterns, which explicitly incorporate information on protein–ligand interactions.^22–25^ However, most of these approaches do not consider how the generated compounds could be synthesized, hindering their transferability to bench experiments.^26^ The synthetic accessibility (SA) score^27^ remains widely used for calculating synthetic feasibility; this heuristic evaluates viability on the basis of the partial structural similarity of the tested compound to known compounds and is effective for high-throughput and coarse assessments. However, it cannot precisely evaluate synthetic reliability.^28^ Retrosynthesis models^29–31^ generate putative routes from commercially available starting materials by iteratively decomposing molecules into reactants; however, they continue to face challenges with regard to capturing reaction selectivity, where expert knowledge and experience remain indispensable.^32,33^

To address this issue, molecular generative models that explicitly incorporate chemical reactions have been developed. Traditionally, exhaustive screening procedures have been performed on vast virtual libraries^34,35^ constructed through virtual combinatorial synthesis using predefined reaction rules (reaction templates) and building blocks. In recent years, however, models to directly optimize objective functions have been developed, and they are expected to more efficiently identify compounds with desired physicochemical properties. Regarding rule-based molecular generation models, methods have been developed that utilize reaction templates describing structural transformations from reactants to products to efficiently generate compounds with high objective function values.^36–38^ In recent years, deep learning-based approaches have been proposed for generating molecules based on reaction templates. The early studies focused on generating compounds that were similar to reference molecular fingerprints.^39,40^ More advanced methods embed sequences of reactants, reaction templates, and products into latent spaces^41,42^ or employ a product-of-experts (PoE) framework to explore the compound space while anchoring a pretrained model on reaction template-derived chemical spaces.^43^ For more directly optimizing the target properties, reinforcement learning-based methods^44,45^ have been developed; these methods train neural networks to select both the reaction templates and building blocks (reactants) that maximize the rewards associated with the desired property values.

However, reinforcement learning approaches excessively prioritize modes that maximize rewards, potentially compromising the diversity of the generated compounds. To address this limitation, the recently proposed generative flow networks (GFlowNet)^46,47^ train models to sample data points with probabilities that are proportional to their reward values. This approach enables the stochastic generation of not only high-reward compounds but also compounds with moderate to low rewards. By avoiding local optima, this method facilitates the discovery of diverse candidate molecules. Recent years have seen rapid progress in terms of the application of GFlowNet to small-molecule generation tasks, with notable achievements in fields such as Bayesian optimization,^48^ genetic algorithms,^49^ multiobjective optimization,^50^ and conditional generation for target proteins.^51^ Methods that apply GFlowNet to reaction template-based compound exploration scenarios^52–54^ have demonstrated the ability to produce candidate compounds with higher reward values and greater diversity than those yielded by reinforcement learning baselines. Most recently, 3DSynthFlow^55^ was proposed as a structural generation method that integrates GFlowNet to sequentially add building blocks and refine their conformations.

The molecular generation models discussed thus far share a common limitation: they all rely on reaction templates or simple compound decomposition rules. Such methods implicitly assume that the structural transformations encoded in the reaction templates proceed as written. However, these simple rules have an inherent inability to fully capture the crucial aspects of the selectivity of chemical reactions. In principle, template descriptions can be refined to address aspects such as chemo- and regioselectivity. However, this leads to a prohibitive expansion in the number of templates, making it impractical to keep pace with the continual emergence of new chemical reactions that are reported daily.

Molecule Chef^56^ and DoG-Gen^57^ employ explicit learning approaches for chemical reaction data sets, offering potential solutions to the limitations of using reaction templates. While these models have enabled the generation of structures via pathways that more closely resemble real-world chemical reactions through reaction prediction, they have focused primarily on learning the distributions of training data sets via maximum likelihood estimation and lack direct optimization frameworks for compound property values. To address this limitation, TRACER^58^ was proposed; this approach combines a transformer-based model for chemical reaction learning with direct optimization through a Monte Carlo tree search (MCTS). The direct optimization scheme of TRACER allows it to explore a broader chemical space than that of the training data set while still incorporating real-world chemical reaction data. Unlike other methods, TRACER supports structure generation in a hit-to-lead manner by specifying a starting compound.

However, TRACER, which combines deep learning models with an MCTS, can be classified as a form of reinforcement learning. Consequently, it faces the same limitations as those of other reinforcement learning-based models: the search process tends to focus excessively on high-reward modes, leaving room for improvement in terms of generating diverse molecules. In this study, we propose TRACE-GFN, which integrates the diverse data sampling capabilities of GFlowNet with the explicit learning method of TRACER for chemical reactions. The objective of this approach is to generate reaction pathways that account for real-world chemical reactions while efficiently exploring diverse compounds.

## Methods

### Overview

The overall architecture of this research is illustrated in Figure 1. In TRACE-GFN, the molecular generation task with reaction pathway generation is formalized as a Markov decision process (MDP) based on TRACER,^58^ with GFlowNet employed to sample reaction trajectories with probabilities that are proportional to the reward function. In TRACER, reactants are input into a graph convolutional network (GCN) to predict applicable reaction templates, which are then conditionally used for product prediction (Figure 1a). By combining this scheme with the GFlowNet framework, a recursive process is implemented to generate molecules (Figure 1b). At each step, the state *s*_*i*_ represents the current reactants (in the SMILES format), while the action *a* denotes the applied reaction template. The GCN models the policy π­(*a*|*s*) to output a distribution of candidate reaction templates. A conditional transformer then approximates *T*(*s*_*i*+1_|*s*_*i*_,*a*) to predict the product SMILES under the specified reaction template. The resulting products are subsequently evaluated by a quantitative structure–activity relationship (QSAR) model and receive a scalar reward *R*(*x*).

**Figure 1:**
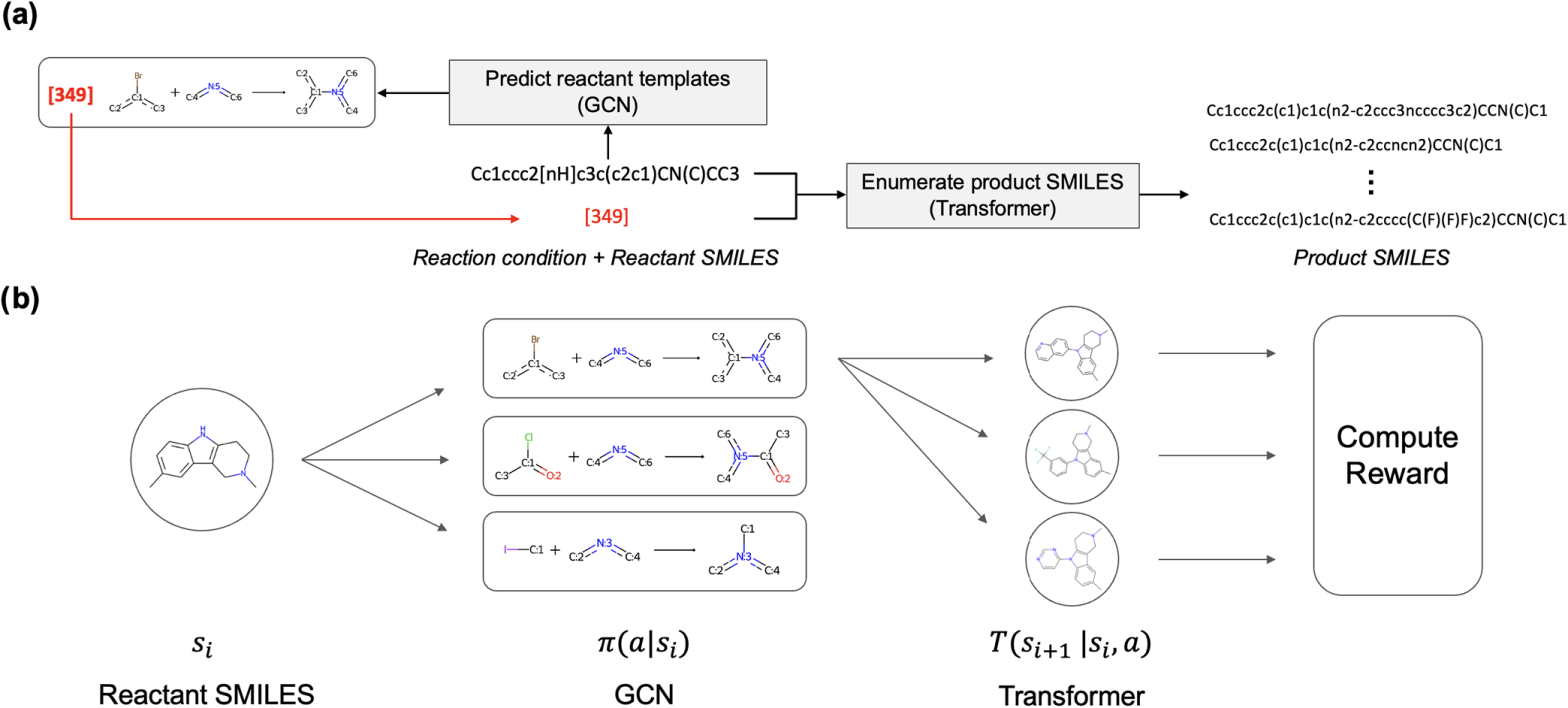
Overview of the molecular generation framework. (a) The model takes reaction conditions and reactant SMILES as inputs. A GCN predicts an index of reaction templates, and a conditional transformer enumerates the possible product SMILES. The index of the reaction template is added to the beginning of reactant SMILES as a conditional token. (b) The generative flow is formulated as a Markov decision process (MDP). Given the current state *s*_*i*_ (reactant SMILES), the GCN policy π­(*a*|*s*_*i*_) predicts reaction templates, and the transformer transition model *T*(*s*_*i+1*_|*s*_*i*_,*a*) generates corresponding product structures. The generated molecules are then evaluated through a reward function, guiding the learning process of the GFlowNet.

### TRACER

TRACER^58^ employs GCN and conditional transformer models for forward reaction prediction and uses a MCTS to search for high reward compounds while executing virtual multistep reactions. In this model, the GCN predicts applicable reaction templates from the reactants, while the conditional transformer enumerates product SMILES sequences under the reaction type constraints.

#### GCN

To select the appropriate reaction templates for a given reactant, the GCN predicts the probability distribution of the applicable reaction templates from the input SMILES of the reactant. Specifically, the architecture consists of stacked basic blocks, i.e., graph convolution → batch normalization → ReLU (rectified linear unit) activation function, over the reactant molecular graph, followed by graph representation aggregation implemented through sum pooling and subsequent processing performed through a fully connected layer that outputs a probability distribution over different chemical reaction classes. The reactants and their corresponding ground-truth reaction templates are sourced from the USPTO (United States Patent and Trademark Office) 1k TPL data set,^59^ which contains 1,000 distinct reaction templates. The detailed mathematical explanation of the graph convolution, dense, and aggregation layers are provided in the Supporting Information.

The hyperparameters of the GCN model were determined via Optuna,^60^ with the hidden-layer dimensionality set to 256, 1 convolutional layer, 3 dense layers, and a learning rate of 0.0004. The final evaluation of the trained GCN model performed on test data yielded the following performance metrics: top-1 accuracy, 0.483; top-5 accuracy, 0.771; and top-10 accuracy, 0.862.^58^

#### Conditional Transformer

In the transformer component of TRACER, conditional generation is achieved by prepending a condition token to the input sequence. As shown in Figure 1a, tokens representing the reaction templates predicted by the GCN are prepended to the reactant SMILES strings. The forward synthesis model is then trained under these constraints. A key feature of TRACER is that it directly generates products while controlling the reaction types using a template index, assuming that multiple possible reactions can proceed from a single starting material. Even when reactant partners exist, they are implicitly learned and identified through the template and structural differences between the reactants and products.^58^

Both the conditional transformer and the GCN used in TRACE-GFN underwent pretraining using the same methodology as that employed for TRACER. The training data set, i.e., the USPTO 1k TPL data set,^59^ comprised 445,000 reactions extracted from the USPTO-sourced reaction database published by Lowe,^61^ paired with their corresponding templates. The data set was filtered to retain only the top 1,000 most frequent templates, which were selected on the basis of atom mapping and template extraction processes performed by RxnMapper. For preprocessing, common reagents and solvents such as hydrochloric acid, ethyl acetate, and dichloromethane were removed, and for each reaction, the molecular pairs that maximized the Tanimoto similarity between the reactants and products were created. The hyperparameters of the conditional transformer were configured according to the original article:^62^ a batch size of 128, a model dimensionality of 512, a dropout rate of 0.1, 6 layers each for the encoder and decoder, 2048 dimensions for the feedforward network, a ReLU activation function, and 8 attention heads for the multihead attention mechanism. The conditional transformer was pretrained on an Ubuntu 22.04 system equipped with a Xeon Gold 5318Y processor, 128GB of RAM, and an RTX 4090 GPU (24GB of VRAM). Detailed information about the pretraining process, parameters, and code is provided in the original paper.^58^

### TRACE-GFN

#### GFlowNet

A GFlowNet is employed as a probability model that generates samples proportional to an unnormalized reward function *R*(*x*) > 0, where the data point *x* serves as the molecular representation. Here, the state space (the set of all possible states) is defined as *S*, and the set of directed transitions (actions) between states is defined as *A*, forming a directed acyclic graph (DAG) *G* = (*S*, *A*). Let *s*_0_ ∈ *S* denote the starting point (initial state), and let *X* ⊂ *S* denote the set of terminal states corresponding to molecules. An action *a* ∈ *A* enables movement from state *s* to *s*′ with a transition probability of *T*(*s*′|*s*,*a*), where *T*(·) denotes the one-step transition probability when action *a* is taken.

The complete trajectory leading up to a terminal state *x* ∈ *X* is denoted as shown in eq 1.

1 $$\tau =\left(s_{0}\to s_{1}\to \cdot \cdot \cdot \to s_{T}=x\right)$$

GFlowNet learns the trajectory distribution using the forward policy *P*_*F*_(*s*_*t*+1_|*s*_*t*_;θ) shown in eq 2, where θ represents the policy parameters, while simultaneously learning an estimator for the proportionality constant (partition function) *Z*_θ_ > 0.

2 $$P_{F}\left(\tau ;\theta \right)=\prod_{t=0}^{T-1}P_{F}\left(s_{t+1}|s_{t};\theta \right)$$

For training, the trajectory balance (TB) objective function^63,64^ is employed. For any complete trajectory τ = (*s*_0_ → ··· → *s*_*T*_ = *x*), the TB loss is given by eq 3.

3 $$L_{\mathrm{TB}}\left(\tau \right)={\left(\log\frac{Z_{\theta }\prod_{t=0}^{T-1}P_{F}\left(s_{t+1}|s_{t};\theta \right)}{R\left(x\right)\prod_{t=1}^{T}P_{B}\left(s_{t-1}|s_{t}\right)}\right)}^{2}$$

Here, *P*_*B*_(*s*_*t*–1_|*s*_*t*_) represents the backward policy, which we define in this study as a uniform distribution over the set of parent states Pa­(*s*_*t*_) = {*u* ∈ *S*|(*u* → *s*_*t*_) ∈ *A*} for each state *s*_*t*_. The uniform *P*_*B*_ is widely used as a simple and computationally efficient approximation in domains where explicitly modeling the parent distributions is challenging.^51,54^

#### Integration of TRACER with GFlowNet

In this study, the state *s* was modeled as a molecule and the action *a* was modeled as a reaction template. To predict the reaction templates, a policy network π_θ_(*a*|*s*) (where θ represents the parameters of the GCN) was used. Furthermore, the transition from a state *s* to *s*′ was treated as a chemical reaction, and the transition probability *T*_ϕ_(*s*′|*s*,*a*) to the product state *s*′ given the reactant compound and the selected reaction template was represented using a conditional transformer model (with its parameters denoted by ϕ). As a result, the forward policy is defined by eq 4, where *a*_*t*_ denotes the action selected at time step *t*.

4 $$P_{F}\left(s_{t+1}|s_{t}\right)=\sum_{a_{t+1}}\pi_{\theta }\left(a_{t+1}|s_{t}\right)T_{\phi }\left(s_{t+1}|s_{t},a_{t+1}\right)$$

In accordance with previous research,^46,53^ a temperature parameter β for the reward function *R*(*x*) was introduced to train models with a probability proportional to *R*(*x*)^β^. By adjusting β, the trade-off between achieving higher rewards and maintaining the sample diversity generated by the forward policy *P*_*F*_ could be controlled. Reflecting these considerations, the trajectory balance objective function is given by eq 5.

5 $$L_{\mathrm{TB}}\left(\tau \right)={\left(\log\frac{Z_{\theta }\left(\beta \right)\prod_{t=0}^{T-1}\pi_{\theta }\left(a_{t+1}|s_{t}\right)T_{\phi }\left(s_{t+1}|s_{t},a_{t+1}\right)}{R{\left(x\right)}^{\beta }\prod_{t=1}^{T}P_{B}\left(s_{t-1}|s_{t}\right)}\right)}^{2}$$

Here, *Z*_θ_(β) is the temperature-dependent estimator for the partition function. In this study, *Z*_θ_(β) was modeled using a multilayer perceptron with three hidden layers.

#### Training of TRACE-GFN

In the TRACE-GFN approach, pretrained GCN and transformer models were employed as initial parameter values for the model. Trajectories were generated by executing multistep chemical reactions. Since TRACE-GFN defines chemical reactions as state transitions, all the resulting states constitute valid molecules. Therefore, every generated compound was evaluated using the reward function *R*(*x*), with all the compounds classified as valid trajectories.

For trajectory generation purposes, the rule-based structural generation model DOGS^36^ was adapted for chemical reactions and was implemented as follows:

1. A GCN was utilized to predict applicable reaction templates for the starting compound.
2. The reaction templates were sampled on the basis of the probabilities predicted by the GCN.
3. Products were generated for the selected reaction using a beam search.
4. The above process was repeated until the predefined maximum trajectory length was reached.

In this study, the maximum reaction length was set to 5, and trajectories were sampled by repeating the above process for 100 rounds with a batch size of 64 and a beam width of 50. These generated structural trajectories with chemical reaction pathways were utilized to train the GCN, conditional transformer, and multilayer perceptron using the trajectory balance objective function.

Both the GCN and transformer components were trained simultaneously using the trajectory balance loss.^63,64^ The computing environment for the experiments was an Ubuntu 22.04 OS, an Intel­(R) Xeon­(R) Gold 5318Y CPU, 128GB of RAM, and an NVIDIA V100 GPU (with 32GB of memory). Using a single GPU under the experimental conditions of this study (maximum reaction length of 5, 100 sampling rounds, batch size of 64, beam width of 50), training of TRACE-GFN required approximately 70 h on average across all starting materials. The majority of the computational cost is attributed to trajectory sampling including forward reaction prediction via the transformer. The computational cost increases with both the maximum reaction length and the beam width. When using larger chemical reaction data sets, scaling up the transformer model may be necessary to accommodate the diversity of reactions. Since trajectories are sampled independently, parallelization across multiple GPUs is expected to reduce calculation time.

### QSAR Model

As in TRACER,^58^ predictive models for dopamine receptor D2 (DRD2), AKT serine/threonine kinase 1 (AKT1), and C-X-C motif chemokine receptor 4 (CXCR4) were employed as evaluation functions for optimization. Utilizing the active/inactive labeled data sets collected from Directory of DUD-E,^65^ ExCAPE^66^ and ChEMBL,^67^ random forest classifiers were trained to predict the activity of compounds from their extended connectivity fingerprints (ECFPs). The composition of the training data set was as follows: 9,006 active compounds and 306,457 inactive compounds for DRD2; 3,623 active compounds and 14,814 inactive compounds for AKT1; and 853 active compounds and 3,051 inactive compounds for CXCR4. The hyperparameters of these models were optimized using Optuna,^60^ with detailed specifications provided in TRACER.^58^

It should be noted that QSAR models have an inherent applicability domain limitation;^68^ their predictive performance may decrease for compounds that are far from the chemical space of the training data set. Molecular generative models often generate structures that are not included in the training data, and thus the calculated QSAR values have an intrinsic uncertainty. In this framework, the QSAR values are used primarily to prioritize compounds generated by the model, rather than to guarantee their biological activity. Complementary computational assessment and experimental validation remain essential to confirm the activity of the generated compounds.

### Selection of Starting Materials

The starting materials used in the molecular structure optimization experiments were identical to those used in previous work.^58^ The selected starting compounds are shown in Figure 2. They were randomly selected from the QSAR value distributions of DRD2, AKT1, and CXCR4. The USPTO 1k TPL data set^59^ was filtered to remove molecules containing rings with eight or more members or molecular weights exceeding 300. Additionally, molecules lacking reactive functional groups (halogen atoms, carbonyl groups, unsaturated bonds, or nucleophilic substituents) were also excluded. Subsequently, for each target protein, the QSAR values of the filtered compounds were calculated, and starting compounds were randomly selected from each QSAR value range: 0–0.1, 0.1–0.2, 0.2–0.3, 0.3–0.4, and 0.4–0.5.

**Figure 2:**
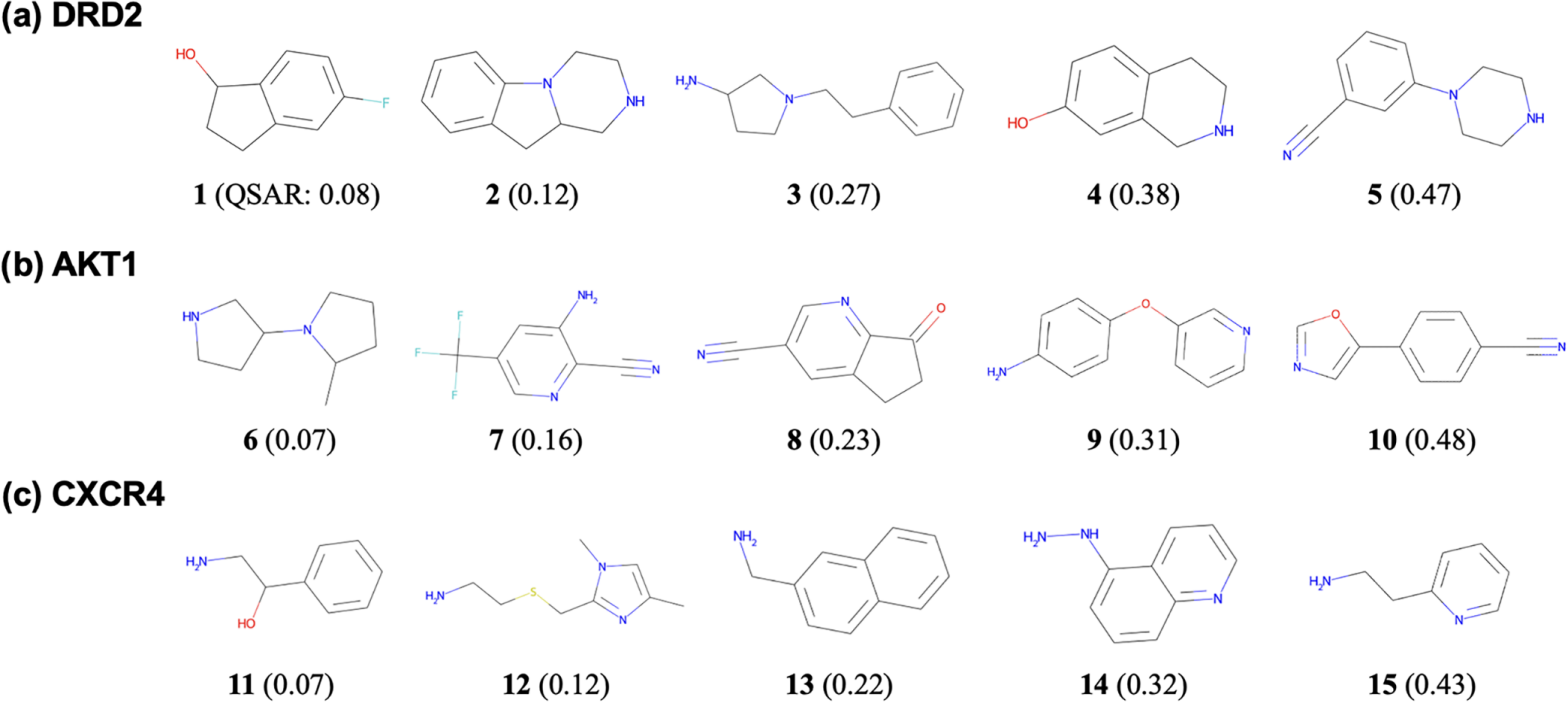
Starting compounds for three targets. (a) DRD2, (b) AKT1, and (c) CXCR4. For each target, five representative starting compounds are shown. One compound was selected from each of the five bins on the basis of target-specific QSAR scores: 0.0–0.1, 0.1–0.2, 0.2–0.3, 0.3–0.4, and 0.4–0.5. Parentheses denote the QSAR values.

## Results and Discussion

### Effect of the Temperature Parameter

The distribution of the compounds generated using the [0,1] output range of the DRD2 activity prediction model as the reward function *R*(*x*) was investigated while the temperature parameter β of the TRACE-GFN reward was varied to 8, 16, and 32. As a representative starting compound, **1** was selected according to the procedure described in the Methods section. For each β, a model was trained and sampling was performed with the trained model. The total number of generated compounds and their property distributions are summarized in Table 1. The diversity and rewards of the generated compounds were evaluated. Here, diversity was calculated as Diversity = 1 – Similarity, where “similarity” denotes the Tanimoto similarity computed by conducting pairwise comparisons within the compound set.

**Table 1:** Comparison among the Rewards and Diversity Levels Achieved in Different Temperature Experiments

		reward		diversity	
temperature	total	mean	std	mean	std
8.0	8525	0.177	0.132	0.710	0.120
16.0	6348	0.571	0.191	0.641	0.116
32.0	7230	0.635	0.192	0.571	0.109

When the temperature parameter was increased from 8 to 32, the reward distribution shifted toward higher values (Figure 3a). This tendency reflects the weighting effect whereby compounds with higher reward values are increasingly favored by *R*(*x*)^β^ as β increases. In contrast, the diversity level tended to decrease with increasing temperature (Figure 3b). These results demonstrate that, in the trajectory balance objective function, the reward values are scaled by *R*(*x*)^β^, where β controls the trade-off between the exploitation of compounds with high reward values and the exploration of the chemical space.

**Figure 3:**
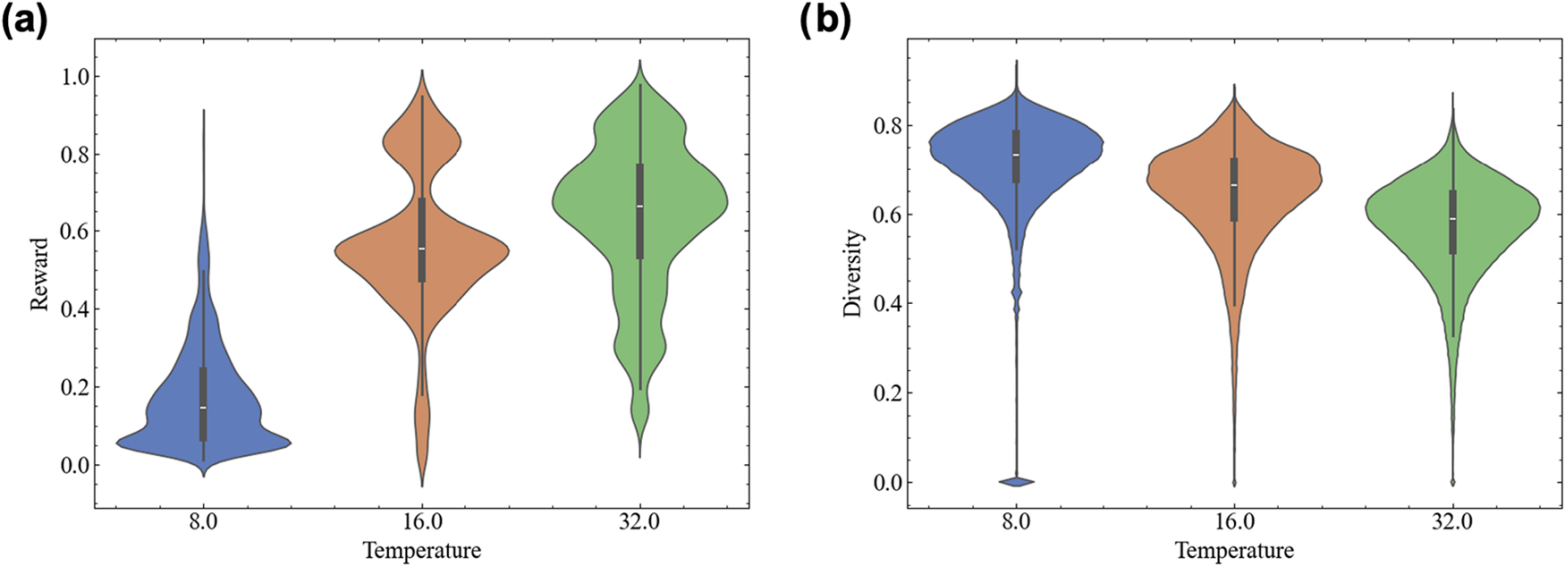
Distribution of the molecules generated across different temperature (β) values. (a) Reward: Distribution of the output values derived from the DRD2 activity QSAR model. (b) Diversity: Internal diversity (1 – Tanimoto similarity) distribution based on ECFP4 (radius 2, 2048 bits). Each violin plot of (a, b) represents the set of molecules generated under conditions with β = 8, 16, 32, with the inner box-and-whisker plot indicating the median and interquartile range.

### Reward Distribution for Each Starting Material

To assess how TRACE-GFN is influenced by the selected starting materials, the reward distributions of products generated from five randomly selected DRD2 starting compounds (**1**–**5**) with QSAR scores falling within specific bins (0–0.1, 0.1–0.2, 0.2–0.3, 0.3–0.4, and 0.4–0.5; see the Methods section) were compared. Figure 4 shows the distribution of the DRD2 activity values predicted for the molecules generated from each starting compound under identical protocol conditions. Considering the temperature examination conducted in the previous section, to balance the reward values with the diversity level, the temperature parameter for TRACE-GFN was set to β = 16.

**Figure 4:**
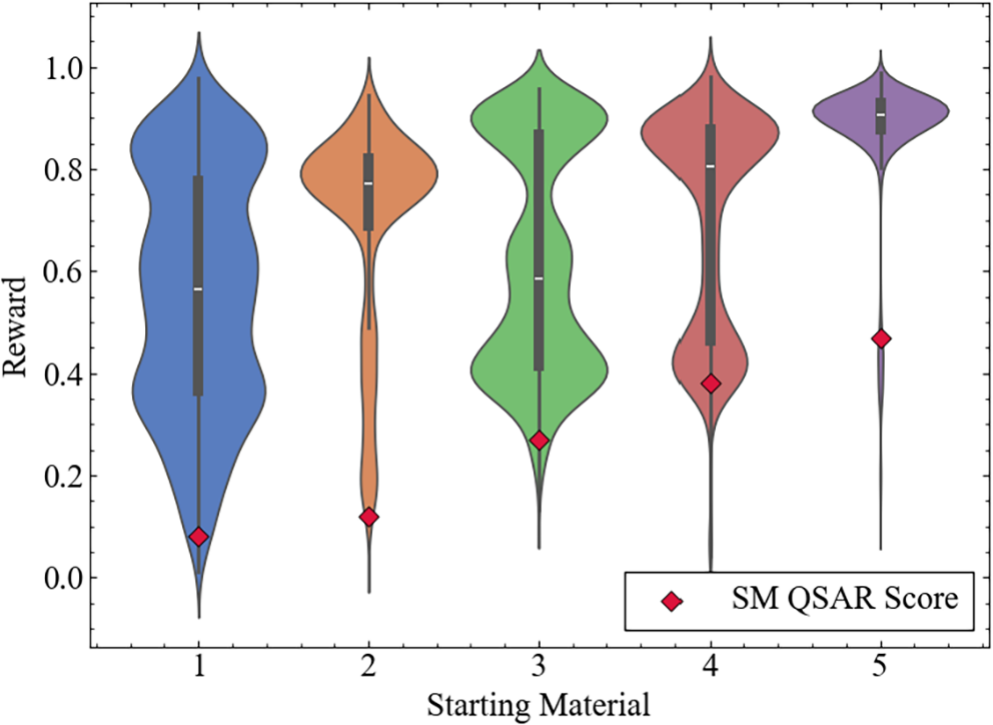
Distributions of the rewards produced for compounds generated using the QSAR model of DRD2 as the reward function (β = 16) for starting materials (SMs) **1**–**5**.

The reward distribution of starting compound **1** exhibited the widest range, with moderate efficiency in terms of producing compounds with high reward values. This likely stemmed from the selection of a compound from the low-QSAR bin (0–0.1), which did not originate from a promising scaffold. In contrast, starting compounds **2** and **5** efficiently produced compounds with high reward values. For **2**, despite the initial QSAR value of 0.12 being relatively low, the chemical reactions performed by TRACE-GFN successfully identified structurally active configurations, which were subsequently sampled by GFlowNet with a high probability. For **5**, the high QSAR value of the starting material provided an advantage by enabling the selection of a promising scaffold. For compounds **3** and **4**, two distinct peaks were observed: one located at moderate reward values and another at high reward values. This distribution pattern suggests that while the initial exploration procedure struggled to identify high-reward compounds, increasing the number of steps enabled the system to locate and focus the exploration on hot spots, resulting in a bimodal distribution. These results indicate that although TRACE-GFN is influenced to some extent by the starting material, it maintains the ability to discover compounds with high reward values across diverse starting compounds.

### Benchmarking TRACE-GFN across Different Protein Targets

A comparative analysis of TRACE-GFN with the conventional methods was conducted via a drug design procedure that incorporated synthetic pathways for multiple protein targets. For the three protein targets (DRD2, AKT1, and CXCR4), structural optimization was performed using target-specific QSAR models as rewards. These proteins were selected to assess the robustness of the developed model with respect to the number of known active ligands, for which 9,006, 3,623, and 853 active ligands have been reported, respectively.

#### Molecular Generation Models Employed in the Comparison

For benchmarking purposes, molecular generation models capable of explicitly learning chemical reactions were selected. TRACE-GFN and TRACER support the specification of an initial compound. In contrast, Molecule Chef and DoG-Gen do not support this feature, enabling the exploration of a broader range of compounds; however, a potential drawback of this approach is that undesirable initial compounds may be selected.

##### TRACER

TRACER served as the predecessor to our method, employing the same GCN and conditional transformer architecture. The key difference between TRACER and TRACE-GFN lies in the integration of a model trained on the USPTO 1k TPL data set with a MCTS. Multistep reactions were performed through the MCTS while searching for compounds with high reward values but without updating the model parameters.^58^

##### Molecule Chef

Molecule Chef represents one of the earliest reported molecular generation models that is capable of learning single-step chemical reactions.^56^ The reactants involved in chemical reactions are converted into “bags of reactants” via graph neural network (GNN)-based vectorization and summation processes, and an encoder–decoder system is then trained to map these outputs to products. The original paper implemented molecular optimization by updating the latent coordinates to maximize the value of a property predictor connected to the latent space. For the comparative experiments conducted herein, precomputed QSAR values from the original training data set (derived from the USPTO data set) were used.

##### DoG-Gen

DoG-Gen was the first molecular generation model to enable the learning of multistep chemical reactions.^57^ DoG-Gen uses two GNN architectures: one for learning molecular structures with atoms as nodes and bonds as edges and another for training reaction trees with molecules as nodes and reactions as edges. Molecular optimization is achieved through sampling using the trained model, with compounds possessing high reward values selected for fine-tuning. In the benchmark experiments, 30 optimization rounds were conducted; in each round, 7,000 reaction trees were generated with DoG-Gen, and the model was fine-tuned twice using the 1,500 top-ranked molecules. A data set derived from the same USPTO corpus as that employed in the original paper was used.

The differences among these models are summarized in Table 2. TRACE-GFN is designed to learn chemical reactions explicitly, supporting molecular generation from specific starting materials, while also enabling the exploration of diverse molecules via the GFlowNet framework. For TRACE-GFN and TRACER, a starting compound must be specified, with compounds randomly selected from the QSAR value bins for each target (0–0.1, 0.1–0.2, ..., 0.4–0.5; see the Methods section). To compare the distributions of the molecules generated by Molecule Chef and DoG-Gen with those of TRACE-GFN and TRACER, the models were evaluated using combined data for the compounds generated from five different starting compounds.

**Table 2:** Comparison of Reaction-Aware Molecular Generative Models^a^

model	optimization method	specification of starting material	multistep reaction
TRACE-GFN	GFlowNet (TB loss)	√	√
TRACER	MCTS	√	√
DoG-Gen	Sampling and fine-tuning	×	√
Molecule Chef	Property predictor	×	×

a Each model is compared in terms of the optimization method, the capability to support multi-step reactions, and the ability to specify a starting material.

#### Evaluation Metrics

For the benchmarking experiments, the following evaluation metrics were computed to assess the compounds generated by each model: (i) the total number of generated compounds (Total); (ii) the proportion of unique compounds (uniqueness); (iii) the proportion of unique compounds with respect to the training data set (uniqueness to USPTO); (iv) the Fréchet ChemNet distance (FCD),^69^ which quantifies the distance between the distributions of the generated compounds and the training data set; (v) the number of compounds with QSAR values ≥0.5; and (vi) diversity. For the comparative experiments, we used the internal diversity measure proposed in the benchmark study on molecular generation models,^70^ which is given by eq 6.

6 $$\mathrm{IntDiv}\left(G\right)=1-\sqrt{\frac{1}{|G{|}^{2}}\sum_{m_{1},m_{2}\in G}T{\left(m_{1},m_{2}\right)}^{2}}$$

Here, *G* denotes the set of generated molecules, and *T*(*m*_1_, *m*_2_) denotes the Tanimoto similarity between molecules *m*_1_ and *m*_2_. To evaluate the synthetic feasibility of molecules generated by each model, the SA score^27^ as implemented in RDKit^71^ was calculated. The SA score ranges from 1 (synthetically feasible) to 10 (synthetically challenging).

#### Experimental Results

The experimental results obtained for the comparison between our method and the existing approaches on DRD2, AKT1, and CXCR4 are shown in Table 3. With respect to the compound generation efficiency achieved for compounds with high QSAR values, TRACE-GFN outperformed all existing methods across all the targets. The proportions of compounds with QSAR values ≥0.5 generated by TRACE-GFN were 48.6%, 71.5%, and 62.6% for DRD2, AKT1, and CXCR4, respectively, surpassing those generated by TRACER, Molecule Chef, and DoG-Gen. In terms of diversity, DoG-Gen had the highest values for AKT1 and CXCR4, whereas TRACE-GFN outperformed TRACER across all the targets. The greater diversity of DoG-Gen can be attributed to its lack of specified starting materials, allowing for greater freedom during compound generation. Comparisons between TRACE-GFN and TRACER demonstrated that TRACE-GFN exhibited improved efficiency in terms of generating compounds with high reward values through model parameter updates while simultaneously producing diverse compounds via GFlowNet.

**Table 3:** Comparison among the Results Produced by the Molecular Generation Models for Each Protein^a^

						QSAR > 0.5	
protein	model	total	uniqueness (%)	uniqueness to USPTO (%)	FCD	num	diversity
DRD2	TRACE-GFN (Sum of SMs **1**–**5**)	42,042	71.4	**100**	**30.5**	**20430**(**48.6**%)	**0.548**
	TRACER (Sum of SMs **1**–**5**)	96,043	**89.2**	99.8	14.3	16372(17.0%)	0.523
	Molecule Chef	218,823	80.0	91.1	2.60	1963(0.90%)	0.490
	DoG-Gen	153,199	73.1	41.3	2.97	26780(23.9%)	0.425
AKT1	TRACE-GFN (Sum of SMs **6**–**10**)	44,035	74.6	**100**	**24.3**	**31483**(**71.5**%)	0.605
	TRACER (Sum of SMs **6**–**10**)	40,959	82.3	99.9	12.2	10424(25.5%)	0.564
	Molecule Chef	174,959	**99.5**	95.2	3.13	827(0.47%)	0.534
	DoG-Gen	164,369	74.2	46.2	3.14	34199(28.0%)	**0.639**
CXCR4	TRACE-GFN (Sum of SMs **11**–**15**)	38,206	80.5	**100**	**21.3**	**23921**(**62.6**%)	0.600
	TRACER (Sum of SMs **11**–**15**)	80,280	92.4	99.8	11.2	10834(13.5%)	0.552
	Molecule Chef	151,469	**99.3**	94.1	1.92	765(0.51%)	0.578
	DoG-Gen	143,602	82.6	44.6	4.42	29204(24.6%)	**0.603**

a The evaluation metrics, including uniqueness, FCD, and diversity, are described in the evaluation metrics subsection.

Molecule Chef, which incorporates a property predictor with its latent space, demonstrated limited optimization capabilities. While DoG-Gen achieved moderate success rates with QSAR values ≥0.5, it produced a significant proportion of molecules that overlapped with the USPTO data set. This result likely stemmed from the optimization method that selected high-reward molecules from the compounds generated by DoG-Gen for fine-tuning, only enhancing the likelihood of compounds with high reward values. In contrast, compared with the USPTO data set, TRACE-GFN and TRACER, which dynamically optimized molecules to objective functions, generated molecules with significantly greater uniqueness. These findings indicate that these methods can generate compounds that are unconstrained by the given training data set. Furthermore, the FCD results revealed that TRACE-GFN and TRACER explored structurally distinct compounds beyond those that were present in the training data set, demonstrating their ability to traverse the vast chemical space.

To evaluate the novelty and synthetic ease of the compounds generated by each model, the relationship between the FCD and SA score was analyzed, as illustrated in Figure 5. The FCD metric represents the distance between compounds generated by each model and their training data (the USPTO data set), whereas the SA score denotes the average SA score of the compounds generated in each experiment. When the target protein and generation model were plotted, a trade-off between the FCD and SA score was observed. Both Molecule Chef and DoG-Gen consistently generated compounds with low FCDs and low SA scores. This tendency likely stems from their architectures learning a mapping of the training data distribution, which tends to favor similarity to known compounds, as reflected in the SA score. In comparison, TRACE-GFN and TRACER generated compounds with higher FCD values, indicating a greater proportion of novel compounds. A distinct trade-off between the FCD and SA score was observed among these models, with TRACE-GFN demonstrating a more extensive exploration of the novel compound space at the expense of higher SA scores. This trade-off highlights the essential role of expert chemists in verifying the synthetic accessibility of generated compounds. To further evaluate the synthetic accessibility of the generated molecules, the results of retrosynthetic analysis using AiZynthFinder^72^ are provided in the Supporting Information. It should be noted that the ability to decompose target molecules into commercially available building blocks depends on both the reaction template library and the stock compound list.^73^ In addition, multiobjective optimization experiments were conducted using a reward function that combines the QSAR value with the SA score (*R*(*x*) = *R*_QSAR_(*x*) + λ·*R*_SA_(*x*)), where λ is a weighting coefficient. By incorporating the SA score into the reward function, the average SA score of generated compounds decreased. However, the average QSAR values also decreased, suggesting a trade-off between synthetic ease and QSAR optimization performance (see the Supporting Information for detailed results).

**Figure 5:**
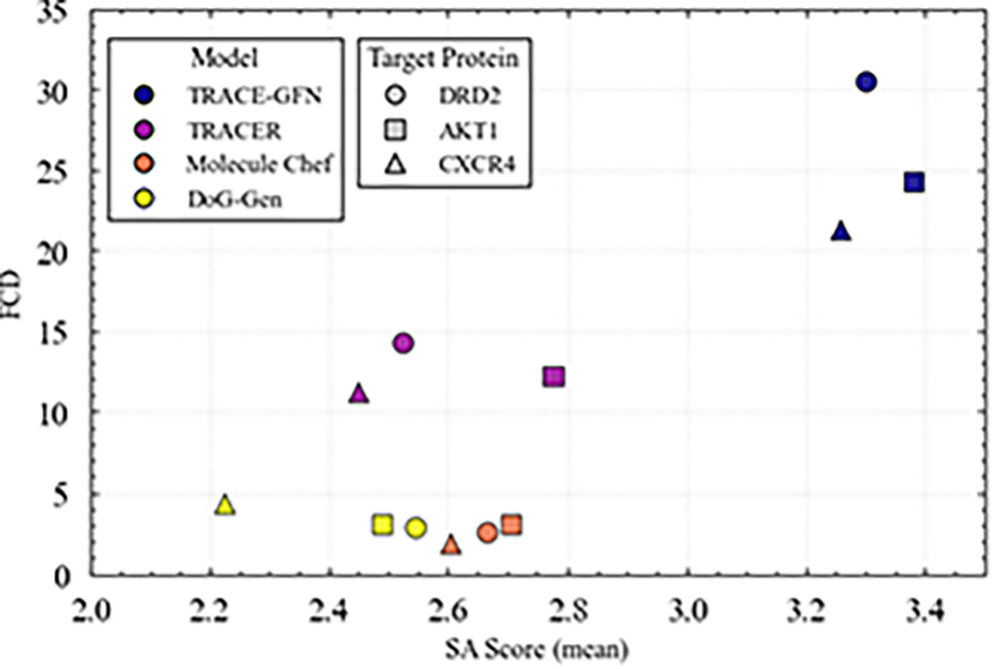
Relationships between the FCD and SA score averages determined for the generated compounds. Each data point represents the calculated metric values for compounds derived from specific targets and generative models. A lower FCD indicates closer alignment with the training distribution (the known chemical space), whereas a lower SA score indicates greater synthetic feasibility. The upper-right area indicates strong novelty but lower synthetic feasibility (high SA score), whereas the lower-left area indicates lower novelty but greater synthetic feasibility.

### Generated Reaction Pathways

TRACE-GFN is capable of generating synthetic pathways while explicitly considering reward values. The highest-reward compounds and their synthetic pathways generated using QSAR models for each target protein are shown in Figure 6. For experiments targeting DRD2, reductive amination and nucleophilic substitution reactions of alkyl halides were selected for starting molecule **4**, resulting in compound **4b** with a QSAR value of 0.980. Previous studies have demonstrated that protecting the phenol group in **4** with triethylsilyl chloride followed by reaction with an acid anhydride enables amide formation at the nitrogen atom.^74^ In experiments targeting AKT1, the selected template for **6** was the substitution reaction with a basic alkyl bromide, similar to a previously reported procedure,^75^ followed by the Suzuki–Miyaura cross-coupling reaction in the absence of competing substituents, yielding compound **6b** with a QSAR value of 0.854 through a simple structural transformation. For experiments targeting CXCR4, aminoalcohol **11** underwent reductive amination with benzaldehyde, Buchwald–Hartwig cross-coupling, and reductive amination of the aldehyde to produce **11c** with a QSAR value of 0.893. While protecting aldehydes or other nucleophilic groups may be necessary for the second and third reaction steps, the first-stage reaction has already been reported to produce **11a** with a 74% yield.^76^

**Figure 6:**
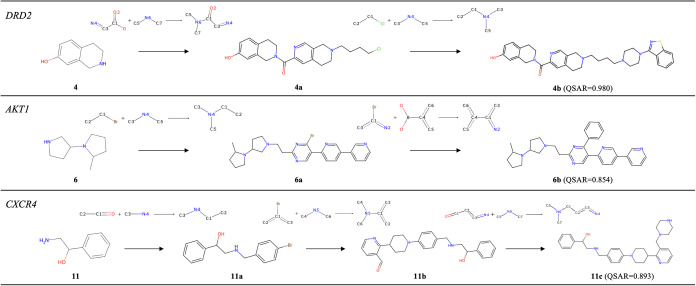
Compounds and synthetic routes generated by TRACE-GFN. For each target protein, the compounds with the highest QSAR prediction values (**4b**, **6b**, and **11c**) and their synthetic routes are shown.

These examples and the results presented in Table 3 demonstrate that TRACE-GFN effectively explores compounds with high reward values through the execution of plausible chemical reactions by learning from real-world chemical reactions. Nevertheless, several limitations should be noted. First, as shown in the synthetic pathway for **11c**, the model does not explicitly control protecting group strategies. In chemical reactions, undesirable side reactions may occur when competing functional groups are present, often requiring selective protection/deprotection steps that the model cannot adequately capture. Second, while the model generates plausible transformations using actual reaction data from the USPTO data set, the model may predict reactions that would not proceed under realistic conditions or generate unintended products. Therefore, the reaction selectivity predicted by the model–including chemoselectivity, regioselectivity, and stereoselectivity–should be regarded as computationally proposed and requires further verification by expert chemists.

## Conclusion

In this study, we propose TRACE-GFN, a combined generative model that incorporates chemical reactions into the drug discovery process by integrating TRACER and GFlowNet. In temperature experiments, it was demonstrated that adjusting this parameter enables an effective balance between exploitation (focusing on generating high-reward compounds) and exploration (widely searching the chemical space) to be achieved. In benchmark experiments targeting three distinct target proteins, TRACE-GFN could robustly optimize the structures of starting compounds with a wide range of QSAR values on the basis of chemical reactions. Moreover, the performance of the TRACE-GFN was superior to that of the other models; TRACE-GFN efficiently generated compounds with high reward values while maintaining strong diversity. Furthermore, the reward function in TRACE-GFN can be replaced with other task-specific predictive models. This means that our framework can be easily extended to molecular design beyond drug discovery, provided that an appropriate reward function is designed for the desired property. Future work is needed to expand the model using larger and more diverse reaction data sets and to apply it to multiobjective optimization frameworks that simultaneously optimize multiple properties.

## Supplementary Material



## Data Availability

The source code is publicly available under an MIT license at https://github.com/sekijima-lab/TRACE-GFN.

## Notes

Retrosynthetic analysis of generated molecules, linear scaling function used to convert the SA score into the synthetic accessibility reward *R*_SA_(*x*), and scatter plot of QSAR values versus SA scores for compounds generated with λ = 0.1 (PDF)

## References

[1] DiMasi J. A., Grabowski H. G., Hansen R. W. (2016). Innovation in the pharmaceutical
industry: new estimates of R&D costs. J.
Health Econ..

[2] Wouters O. J., McKee M., Luyten J. (2020). Estimated Research and Development
Investment Needed to Bring a New Medicine to Market, 2009–2018. JAMA.

[3] Blanco M.-J., Gardinier K. M., Namchuk M. N. (2022). Advancing new chemical modalities
into clinical studies. ACS Med. Chem. Lett..

[4] Hughes J. P., Rees S., Kalindjian S. B., Philpott K. L. (2011). Principles of early
drug discovery. Br. J. Pharm..

[5] Bleicher K. H., Böhm H.-J., Müller K., Alanine A. I. (2003). Hit and lead generation:
beyond high-throughput screening. Nat. Rev.
Drug Discovery.

[6] Yoshino R., Yasuo N., Hagiwara Y., Ishida T., Inaoka D. K., Amano Y., Tateishi Y., Ohno K., Namatame I., Niimi T. (2023). Discovery
of a hidden trypanosoma cruzi spermidine
synthase binding site and inhibitors through in silico, in vitro,
and X-ray crystallography. ACS Omega.

[7] Jiménez-Luna J., Grisoni F., Schneider G. (2020). Drug discovery
with explainable artificial
intelligence. Nat. Mach. Intell..

[8] Weininger D. (1988). SMILES, a
chemical language and information system. 1. Introduction to methodology
and encoding rules. J. Chem. Inf. Comput. Sci..

[9] Sousa T., Correia J., Pereira V., Rocha M. (2021). Generative
deep learning
for targeted compound design. J. Chem. Inf.
Model..

[10] Yang X., Zhang J., Yoshizoe K., Terayama K., Tsuda K. (2017). ChemTS: an
efficient python library for de novo molecular generation. Sci. Technol. Adv. Mater..

[11] Erikawa D., Yasuo N., Suzuki T., Nakamura S., Sekijima M. (2023). Gargoyles:
An open source graph-based molecular optimization method based on
deep reinforcement learning. ACS Omega.

[12] Loeffler H. H., He J., Tibo A., Janet J. P., Voronov A., Mervin L. H., Engkvist O. (2024). Reinvent 4:
Modern AI-driven generative molecule design. J. Cheminf..

[13] Suzuki T., Ma D., Yasuo N., Sekijima M. (2024). Mothra: Multiobjective de novo Molecular
Generation Using Monte Carlo Tree Search. J.
Chem. Inf. Model..

[14] Gómez-Bombarelli R., Wei J. N., Duvenaud D., Hernández-Lobato J. M., Sánchez-Lengeling B., Sheberla D., Aguilera-Iparraguirre J., Hirzel T. D., Adams R. P., Aspuru-Guzik A. (2018). Automatic
Chemical Design Using a Data-Driven Continuous Representation of Molecules. ACS Cent. Sci..

[15] Lim J., Ryu S., Kim J. W., Kim W. Y. (2018). Molecular generative
model based
on conditional variational autoencoder for de novo molecular design. J. Cheminf..

[16] Grechishnikova D. (2021). Transformer
neural network for protein-specific de novo drug generation as a machine
translation problem. Sci. Rep..

[17] Schwaller P., Laino T., Gaudin T., Bolgar P., Hunter C. A., Bekas C., Lee A. A. (2019). Molecular transformer:
a model for
uncertainty-calibrated chemical reaction prediction. ACS Cent. Sci..

[18] Tysinger E. P., Rai B. K., Sinitskiy A. V. (2023). Can We
Quickly Learn to “Translate”
Bioactive Molecules with Transformer Models?. J. Chem. Inf. Model..

[19] Alakhdar A., Poczos B., Washburn N. (2024). Diffusion models in
de novo drug
design. J. Chem. Inf. Model..

[20] Schneuing A., Harris C., Du Y., Didi K., Jamasb A., Igashov I., Du W., Gomes C., Blundell T. L., Lio P., Welling M., Bronstein M., Correia B. (2024). Structure-based drug
design with equivariant diffusion models. Nat.
Comput. Sci..

[21] Lin H., Huang Y., Zhang O., Ma S., Liu M., Li X., Wu L., Wang J., Hou T., Li S. Z. (2025). Diffbp:
Generative diffusion of 3d molecules for target protein binding. Chem. Sci..

[22] Zhang J., Chen H. (2022). De novo molecule design using molecular generative models constrained
by ligand-protein interactions. J. Chem. Inf.
Model..

[23] Ozawa M., Nakamura S., Yasuo N., Sekijima M. (2024). IEV2Mol: Molecular
Generative Model Considering Protein-Ligand Interaction Energy Vectors. J. Chem. Inf. Model..

[24] Zhung W., Kim H., Kim W. Y. (2024). 3D molecular generative
framework for interaction-guided
drug design. Nat. Commun..

[25] Sako M., Yasuo N., Sekijima M. (2025). DiffInt: A diffusion
model for structure-based
drug design with explicit hydrogen bond interaction guidance. J. Chem. Inf. Model..

[26] Schneider G., Clark D. E. (2019). Automated de novo drug design: are
we nearly there
yet?. Angew. Chem., Int. Ed..

[27] Ertl P., Schuffenhauer A. (2009). Estimation
of synthetic accessibility score of drug-like
molecules based on molecular complexity and fragment contributions. J. Cheminf..

[28] Stanley M., Segler M. (2023). Fake it until you make
it? Generative de novo design
and virtual screening of synthesizable molecules. Curr. Opin. Struct. Biol..

[29] Corey E. J. (1991). The logic
of chemical synthesis: multistep synthesis of complex carbogenic molecules
(nobel lecture). Angew. Chem., Int. Ed..

[30] Coley C. W., Barzilay R., Jaakkola T. S., Green W. H., Jensen K. F. (2017). Prediction
of organic reaction outcomes using machine learning. ACS Cent. Sci..

[31] Gricourt G., Meyer P., Duigou T., Faulon J.-L. (2024). Artificial
Intelligence
Methods and Models for Retro-Biosynthesis: A Scoping Review. ACS Synth. Biol..

[32] Bilodeau C., Jin W., Jaakkola T., Barzilay R., Jensen K. F. (2022). Generative models
for molecular discovery: Recent advances and challenges. WIREs Comput. Mol. Sci..

[33] Strieth-Kalthoff F., Szymkuć S., Molga K., Aspuru-Guzik A., Glorius F., Grzybowski B. A. (2024). Artificial
Intelligence for Retrosynthetic
Planning Needs Both Data and Expert Knowledge. J. Am. Chem. Soc..

[34] Hu Q., Peng Z., Sutton S. C., Na J., Kostrowicki J., Yang B., Thacher T., Kong X., Mattaparti S., Zhou J. Z. (2012). Pfizer Global Virtual
Library (PGVL): a chemistry design
tool powered by experimentally validated parallel synthesis information. ACS Comb. Sci..

[35] Grygorenko O. O., Radchenko D. S., Dziuba I., Chuprina A., Gubina K. E., Moroz Y. S. (2020). Generating multibillion chemical space of readily accessible
screening compounds. iScience.

[36] Hartenfeller M., Zettl H., Walter M., Rupp M., Reisen F., Proschak E., Weggen S., Stark H., Schneider G. (2012). DOGS: reaction-driven
de novo design of bioactive compounds. PLoS
Comput. Biol..

[37] Vinkers H. M., de Jonge M. R., Daeyaert F. F., Heeres J., Koymans L. M., van Lenthe J. H., Lewi P. J., Timmerman H., Van Aken K., Janssen P. A. (2003). Synopsis: synthesize and optimize
system in silico. J. Med. Chem..

[38] Spiegel J. O., Durrant J. D. (2020). AutoGrow4: an open-source
genetic algorithm for de
novo drug design and lead optimization. J. Cheminf..

[39] Button A., Merk D., Hiss J. A., Schneider G. (2019). Automated
de novo molecular design by hybrid machine intelligence and rule-driven
chemical synthesis. Nat. Mach. Intell..

[40] Gao, W. ; Mercado, R. ; Coley, C. W. In Amortized Tree Generation for Bottom-up Synthesis Planning and Synthesizable Molecular Design, International Conference on Learning Representations; ICLR, 2022.

[41] Nguyen D. H., Tsuda K. (2022). Generating reaction trees with cascaded variational autoencoders. J. Chem. Phys..

[42] Noh, J. ; Jeong, D.-W. ; Kim, K. ; Han, S. ; Lee, M. ; Lee, H. ; Jung, Y. In Path-Aware and Structure-Preserving Generation of Synthetically Accessible Molecules, Proceedings of the 39th International Conference on Machine Learning; ICML, 2022; pp 16952–16968.

[43] Nakata S., Mori Y., Tanaka S. (2024). Navigating Ultralarge Virtual Chemical
Spaces with Product-of-Experts Chemical Language Models. J. Chem. Inf. Model..

[44] Gottipati, S. K. ; Sattarov, B. ; Niu, S. ; Pathak, Y. ; Wei, H. ; Liu, S. ; Blackburn, S. ; Thomas, K. ; Coley, C. ; Tang, J. In Learning to Navigate the Synthetically Accessible Chemical Space Using Reinforcement Learning, International Conference on Machine Learning; ICML, 2020; pp 3668–3679.

[45] Horwood J., Noutahi E. (2020). Molecular Design in
Synthetically Accessible Chemical
Space via Deep Reinforcement Learning. ACS Omega.

[46] Bengio, E. ; Jain, M. ; Korablyov, M. ; Precup, D. ; Bengio, Y. In Flow Network Based Generative Models for Non-Iterative Diverse Candidate Generation, Advances in Neural Information Processing Systems; NeurIPS, 2021; pp 27381–27394.

[47] Bengio Y., Lahlou S., Deleu T., Hu E. J., Tiwari M., Bengio E. (2023). GFlowNet Foundations. J. Mach.
Learn. Res..

[48] Zhu, Y. ; Wu, J. ; Hu, C. ; Yan, J. ; Hsieh, C.-Y. ; Hou, T. ; Wu, J. In Sample-Efficient Multi-Objective Molecular Optimization with GFlowNets, Thirty-Seventh Conference on Neural Information Processing Systems; NIPS, 2023.

[49] Kim, H. ; Kim, M. ; Choi, S. ; Park, J. In Genetic-Guided GFlowNets for Sample Efficient Molecular Optimization, Thirty-Eighth Annual Conference on Neural Information Processing Systems; NIPS, 2024.

[50] Jain, M. ; Raparthy, S. C. ; Hernández-García, A. ; Rector-Brooks, J. ; Bengio, Y. ; Miret, S. ; Bengio, E. Multi-Objective GFlowNets, 2023. https://openreview.net/forum?id/3z1Ws6GEYV4.

[51] Shen, T. ; Seo, S. ; Lee, G. ; Pandey, M. ; Smith, J. R. ; Cherkasov, A. ; Kim, W. Y. ; Ester, M. In TacoGFN: Target-Conditioned GFlowNet for Structure-Based Drug Design, Transactions on Machine Learning Research; MLR, 2024.

[52] Koziarski, M. ; Rekesh, A. ; Shevchuk, D. ; van der Sloot, A. M. ; Gaiński, P. ; Bengio, Y. ; Liu, C.-H. ; Tyers, M. ; Batey, R. A. In RGFN: Synthesizable Molecular Generation Using GFlowNets, Thirty-Eighth Annual Conference on Neural Information Processing Systems; NIPS, 2024.

[53] Cretu, M. ; Harris, C. ; Roy, J. ; Bengio, E. ; Liò, P. In Synflownet: Towards Molecule Design with Guaranteed Synthesis Pathways, ICLR 2024 Workshop on Generative and Experimental Perspectives for Biomolecular Design; ICLR, 2024.

[54] Seo, S. ; Kim, M. ; Shen, T. ; Ester, M. ; Park, J. ; Ahn, S. ; Kim, W. Y. In Generative Flows on Synthetic Pathway for Drug Design, Thirteenth International Conference on Learning Representations; ICLR, 2025.

[55] Shen, T. ; Seo, S. ; Irwin, R. ; Didi, K. ; Olsson, S. ; Kim, W. Y. ; Ester, M. Compositional Flows for 3D Molecule and Synthesis Pathway Co-design, arXiv:2504.08051. arXiv.org e-Print archive, 2025 https://arxiv.org/abs/2504.08051.

[56] Bradshaw, J. ; Paige, B. ; Kusner, M. J. ; Segler, M. ; Hernández-Lobato, J. M. In A Model to Search for Synthesizable Molecules, Advances in Neural Information Processing Systems; NeurIPS, 2019; pp 12000–12010.

[57] Bradshaw, J. ; Paige, B. ; Kusner, M. J. ; Segler, M. ; Hernández-Lobato, J. M. In Barking Up the Right Tree: An Approach to Search over molecule Synthesis Dags, Advances in Neural Information Processing Systems; NeurIPS, 2020; pp 6852–6866.

[58] Nakamura S., Yasuo N., Sekijima M. (2025). Molecular optimization using a conditional
transformer for reaction-aware compound exploration with reinforcement
learning. Commun. Chem..

[59] Schwaller P., Probst D., Vaucher A. C., Nair V. H., Kreutter D., Laino T., Reymond J.-L. (2021). Mapping the space
of chemical reactions
using attention-based neural networks. Nat.
Mach. Intell..

[60] Akiba, T. ; Sano, S. ; Yanase, T. ; Ohta, T. ; Koyama, M. In Optuna: A Next-Generation Hyperparameter Optimization Framework, Proceedings of the 25th ACM SIGKDD International Conference on Knowledge Discovery & Data Mining; ACM, 2019; pp 2623–2631.

[61] Lowe, D. Chemical reactions from U.S. patents (1976-Sep2016) 2017 https://figshare.com/articles/dataset/Chemical_reactions_from_US_patents_1976-Sep2016_/5104873.

[62] Vaswani, A. ; Shazeer, N. ; Parmar, N. ; Uszkoreit, J. ; Jones, L. ; Gomez, A. N. ; Kaiser, Ł. ; Polosukhin, I. In Attention is All You Need, Advances in Neural Information Processing Systems; NeurIPS, 2017.

[63] Malkin, N. ; Jain, M. ; Bengio, E. ; Sun, C. ; Bengio, Y. In Trajectory Balance: Improved Credit Assignment in GFlowNets, Advances in Neural Information Processing Systems; NeurIPS, 2022; pp 5955–5967.

[64] Madan, K. ; Rector-Brooks, J. ; Korablyov, M. ; Bengio, E. ; Jain, M. ; Nica, A. C. ; Bosc, T. ; Bengio, Y. ; Malkin, N. In Learning GFlowNets from Partial Episodes for Improved Convergence and Stability, International Conference on Machine Learning; ICML, 2023; pp 23467–23483.

[65] Mysinger M. M., Carchia M., Irwin J. J., Shoichet B. K. (2012). Directory
of useful
decoys, enhanced (DUD-E): better ligands and decoys for better benchmarking. J. Med. Chem..

[66] Sun J., Jeliazkova N., Chupakhin V., Golib-Dzib J.-F., Engkvist O., Carlsson L., Wegner J., Ceulemans H., Georgiev I., Jeliazkov V. (2017). ExCAPE-DB: an integrated
large scale dataset facilitating Big Data analysis in chemogenomics. J. Cheminf..

[67] Zdrazil B., Felix E., Hunter F., Manners E. J., Blackshaw J., Corbett S., de Veij M., Ioannidis H., Lopez D. M., Mosquera J. F. (2024). The
ChEMBL Database
in 2023: a drug discovery platform spanning multiple bioactivity data
types and time periods. Nucleic Acids Res..

[68] Cherkasov A., Muratov E. N., Fourches D., Varnek A., Baskin I. I., Cronin M., Dearden J., Gramatica P., Martin Y. C., Todeschini R. (2014). QSAR modeling: where
have you been? Where are you going to?. J. Med.
Chem..

[69] Preuer K., Renz P., Unterthiner T., Hochreiter S., Klambauer G. (2018). Fréchet ChemNet distance:
a metric for generative
models for molecules in drug discovery. J. Chem.
Inf. Model..

[70] Polykovskiy D., Zhebrak A., Sanchez-Lengeling B. (2020). Molecular Sets (MOSES):
A Benchmarking Platform for Molecular Generation Models. Front. Pharmacol..

[71] Landrum, G. RDKit: Open-source cheminformatics, 2026. http://www.rdkit.org, accessed February 21, 2026.

[72] Genheden S., Thakkar A., Chadimová V., Reymond J.-L., Engkvist O., Bjerrum E. (2020). AiZynthFinder: a fast,
robust and flexible open-source
software for retrosynthetic planning. J. Cheminf..

[73] Gao W., Coley C. W. (2020). The synthesizability of molecules proposed by generative
models. J. Chem. Inf. Model..

[74] Möcklinghoff S., Van Otterlo W. A., Rose R., Fuchs S., Zimmermann T. J., Dominguez Seoane M., Waldmann H., Ottmann C., Brunsveld L. (2011). Design and
evaluation of fragment-like estrogen receptor tetrahydroisoquinoline
ligands from a scaffold-detection approach. J. Med. Chem..

[75] Li Z., Wang Y., Fu C., Wang X., Wang J. J., Zhang Y., Zhou D., Zhao Y., Luo L., Ma H. (2018). Design,
synthesis, and structure-activity-relationship
of a novel series of CXCR4 antagonists. Eur.
J. Med. Chem..

[76] Zindell, R. M. ; Riether, D. ; Thomson, D. S. ; Wu, L. ; World Intellectual Property Organization (WIPO) . Compounds which modulate the CB2 receptor. WO Patent, WO2007/118041A1, 2007 https://worldwide.espacenet.com/patent/search/family/038198564/publication/WO2007118041A1. Published Oct 18, 2007.

