# Supplementary material for: A Transformer for Reaction-Aware Compound Explorations with GFlowNet in QSAR-Guided Molecular Design
Source: J Chem Inf Model. 2026 Apr 25;66(9):5101–11. doi: 10.1021/acs.jcim.6c00181 (PMC13169345; doi:10.1021/acs.jcim.6c00181)
Supplement: Supplementary file 1 [file ci6c00181_si_001.pdf]

Supporting Information for A Transformer for Reaction-Aware Compound Explorations with GFlowNet in QSAR-Guided Molecular Design

Shogo Nakamura[1], Nobuaki Yasuo[2], and Masakazu Sekijima[*, 3]

[1]Department of Life Science and Technology, Institute of Science Tokyo, Midori-ku, Yokohama, 226-8501, Japan.
[2]Tokyo Tech Academy for Convergence of Materials and Informatics (TAC-MI), Institute of Science Tokyo, Meguro-ku, Tokyo, 152-8550, Japan.
[3]Department of Computing, Institute of Science Tokyo, Midori-ku, Yokohama, 226-8501, Japan.
[*]Email: sekijima@comp.isct.ac.jp

1 Graph Convolutional Network Architecture

This section provides the mathematical formulations of the GCN used for reaction template prediction. The GCN takes a molecular graph of the reactant as input and outputs a probability distribution over 1,000 reaction templates. The architecture consists of graph convolution layers with batch normalization and ReLU activation, followed by sum pooling for graph-level aggregation and a fully connected layer with softmax output. The specific layer operations are defined as follows:

1. Graph Convolution Layer

   Molecules with n nodes are represented as $\mathcal{M} \equiv (\mathbf{A}, \mathbf{E}, \mathbf{F})$, where $\mathbf{A} \in \{0,1\}^{n\times n}$ is the adjacency matrix, $\mathbf{F} \in \mathbb{R}^{n\times d}$ is the node-feature matrix, and $\mathbf{E} \in \{0,1\}^{n\times n\times T}$ is the edge tensor when a set of bond types $\mathcal{T}$ with $|\mathcal{T}| = T$ is present. When $\mathbf{A} = \{\mathbf{A}^{(t)} \mid t \in \mathcal{T}\}$, the molecule can be written as $\mathcal{M}' \equiv (\mathbf{A}, \mathbf{F})$ with $\mathbf{A}^{(t)}_{ij} = 1$ if a bond of type t exists between nodes i and j. The l-th graph convolution is calculated as follows:

   $$\mathbf{X}^{(l+1)} = \sigma\left(\sum_{t\in\mathcal{T}} \tilde{\mathbf{A}}^{(t)}\,\mathbf{X}^{(l)}\,\mathbf{W}^{(l)}_t\right), \tag{S1}$$

   where $\tilde{\mathbf{A}}^{(t)}$ is a normalized adjacency matrix for bond type t, $\mathbf{X}^{(l)}$ is the input matrix of the l-th layer, $\mathbf{W}^{(l)}_t$ is the parameter matrix for the l-th layer with bond type t, and σ denotes an activation function.

2. Dense Layer

   After the graph convolution process, the output is passed through a nodewise fully connected (dense) layer that independently applies a linear transformation to each node:

   $$\mathbf{X}^{(l+1)} = \mathbf{X}^{(l)}\mathbf{W}^{(l)}. \tag{S2}$$

3. Aggregation Layer

   After the graph convolution and dense layers, the node representations are aggregated to obtain a graph-level representation. Following prior work, sum aggregation is employed as follows:

   $$g = \sum_{i=1}^{n} \mathbf{X}^{(L)}_{i:}, \tag{S3}$$

   where $\mathbf{X}^{(L)}_{i:}$ is the i-th row of the final-layer matrix $\mathbf{X}^{(L)}$. The resulting g is then fed to a classifier with a softmax output to obtain probabilities over the reaction templates (the final layer uses K=1000 outputs for template prediction purposes).

2 Retrosynthetic Analysis using AiZynthFinder

In the manuscript, SA scores and FCD values were used as metrics to evaluate the synthetic accessibility of molecules generated by each model. As a supplementary assessment of synthetic feasibility, an analysis was conducted using AiZynthFinder [1], a

retrosynthesis tool based on reaction templates and a building block list. From the set of compounds generated by each model for their respective target proteins, 1,000 compounds were randomly sampled, and retrosynthetic analyses were performed performed using AiZynthFinder. A result was defined as "solved" when AiZynthFinder successfully proposed at least one synthetic route reaching commercially available starting materials (stock compounds).

For the retrosynthetic searches, a list of reaction templates extracted from the USPTO dataset provided by AiZynthFinder was utilized, with building blocks from ZINC20 [2] serving as the commercially available compounds. It should be noted that the success rate of retrosynthetic evaluation using machine learning-based models are highly influenced by both the dataset of reaction templates and the list of commercially available compounds [3].

The experimental results are summarized in Table S1. DoG-Gen achieved high success rates across all targets. This is likely attributable to its architecture, which learns the distribution of the training dataset. TRACER maintained high success rates since MCTS does not update the parameters of the pre-trained model, allowing the structural transformations to remain close to the learned chemical reactions. Both DoG-Gen and TRACER exhibited high success rates, which may be influenced by AiZynthFinder's use of retrosynthetic analysis using reaction templates extracted from the USPTO dataset. In contrast, TRACE-GFN showed lower success rates since GFlowNet encourages exploration of high-reward regions beyond the distribution of the training dataset. This trend aligns with the tradeoff between FCD and SA scores discussed in the main manuscript. Despite showing the lowest FCD values among all models, Molecule Chef achieved only moderate success rates, exhibiting a different trend from the other methods. One possible explanation is that Molecule Chef may generate compounds with only minor structural modifications from known compounds. Such subtle differences in substituent types or positions could potentially prevent decomposition into commercially available building blocks during retrosynthetic analysis.

Table S1: Retrosynthetic analysis of generated molecules. For each model and target protein, 1,000 compounds were randomly sampled from the generated compound set, and the percentage of compounds for which AiZynthFinder successfully identified at least one retrosynthetic route is reported.

Target	TRACE-GFN	TRACER	DoG-Gen	Molecule Chef
DRD2	45.6%	89.0%	83.0%	56.1%
AKT1	41.0%	77.8%	87.7%	62.3%
CXCR4	43.1%	88.8%	96.5%	60.9%

3 Multi-Objective Optimization with Synthetic Accessibility

As discussed in the main manuscript, a tradeoff between similarity to the training data and synthetic accessibility was observed for each model. To further investigate this tradeoff for TRACE-GFN, multi-objective optimization experiments were conducted by incorporating

the SA score into the reward function. The QSAR model for DRD2 was used as the reward function, and molecule **1** was used as the starting material.

The combined reward function was defined as follows:

$$R(x) = R_{\mathrm{QSAR}}(x) + \lambda \cdot R_{\mathrm{SA}}(x), \tag{S4}$$

where $R_{\mathrm{QSAR}}(x)$ denotes the QSAR value for DRD2 (ranging from 0 to 1), $R_{\mathrm{SA}}(x)$ denotes the scaled SA score reward, and λ is a weighting coefficient that controls the balance between these terms.

The SA score, which ranges from 1 (synthetically feasible) to 10 (synthetically challenging), was transformed into a reward $R_{\mathrm{SA}}(x) \in [0, 1]$ using a linear scaling function:

$$R_{\mathrm{SA}}(x) = \mathrm{clip}\left(\frac{\mathrm{SA}_{\mathrm{max}} - \mathrm{SA}(x)}{\mathrm{SA}_{\mathrm{max}} - \mathrm{SA}_{\mathrm{min}}},\ 0,\ 1\right), \tag{S5}$$

where $\mathrm{SA}_{\mathrm{max}} = 5$ and $\mathrm{SA}_{\mathrm{min}} = 1$ were determined on the basis of the SA score distribution of the compounds generated in the single-objective experiments described in the main manuscript. Under this scaling, compounds with an SA score of 1 receive the maximum reward ($R_{\mathrm{SA}} = 1$), those with an SA score of 5 receive zero reward ($R_{\mathrm{SA}} = 0$), and compounds with SA scores exceeding 5 are clipped to $R_{\mathrm{SA}} = 0$ (Figure S1). Using DRD2 as the target protein and compound **1** as the starting material, four experiments were conducted with $\lambda = 0.0, 0.1, 0.5$, and 1.0. The case of $\lambda = 0.0$ corresponds to the single-objective optimization using only the QSAR reward. The temperature of GFlowNet was fixed at $\beta = 16$ across all experiments.

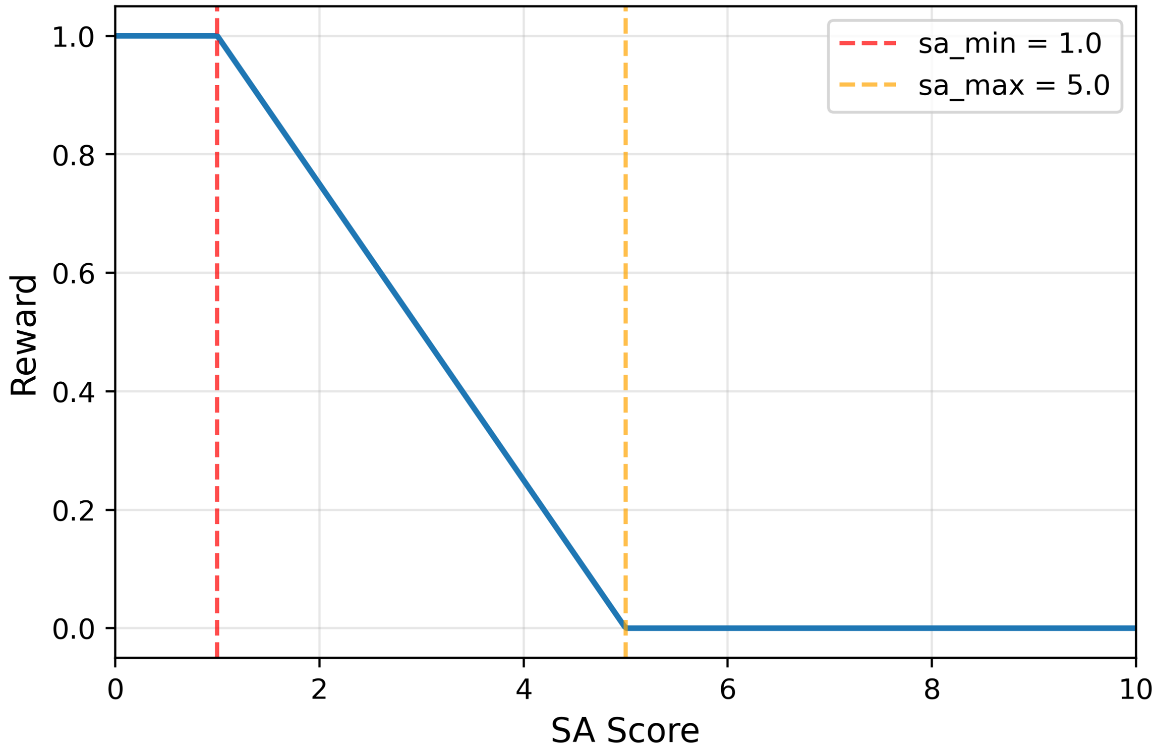

Figure S1: Linear scaling function used to convert the SA score into the synthetic accessibility reward $R_{\mathrm{SA}}(x)$.

The scatter plots of QSAR values versus SA scores for the molecules generated under each λ are shown in Figures S2 – S5. As λ increased, the distribution of generated compounds shifted toward lower SA scores, indicating that more synthetically accessible structures were preferentially sampled. At the same time, the QSAR values decreased with increasing λ, reflecting a tradeoff between QSAR values and synthetic ease. Notably, when λ was small (0.0 or 0.1), the model occasionally generated compounds with high SA scores (4.5–5.5), corresponding to synthetically challenging structures. As λ increased to 0.5 and 1.0, this tendency was substantially suppressed, and the generated compounds were more concentrated in the region of lower SA scores. Furthermore, in experiments conducted with $\lambda = 0.1$ and 0.5, the model generated molecules in hotspot regions characterized by both low SA scores and high QSAR values. Since molecules in these regions possess high reward values, through further training, the model is expected to become more efficient at generating compounds with high QSAR values and low SA scores (i.e., higher synthetic feasibility).

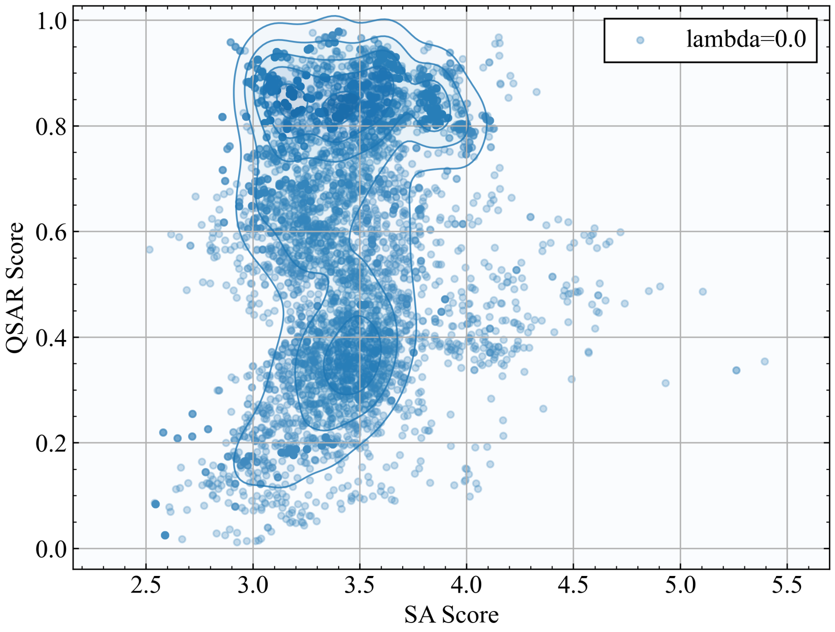

Figure S2: Scatter plot of QSAR values versus SA scores for compounds generated with $\lambda = 0.0$ (QSAR-only optimization).

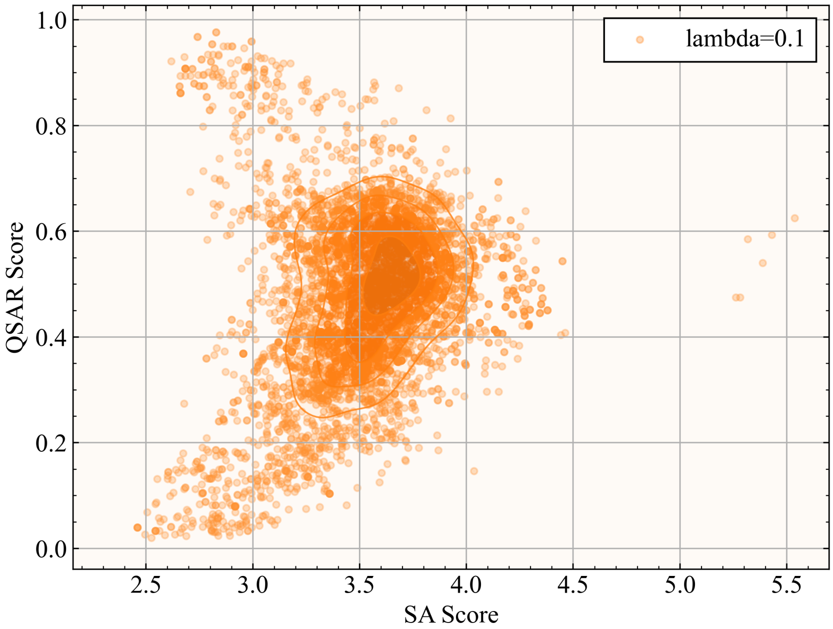

Figure S3: Scatter plot of QSAR values versus SA scores for compounds generated with $\lambda = 0.1$.

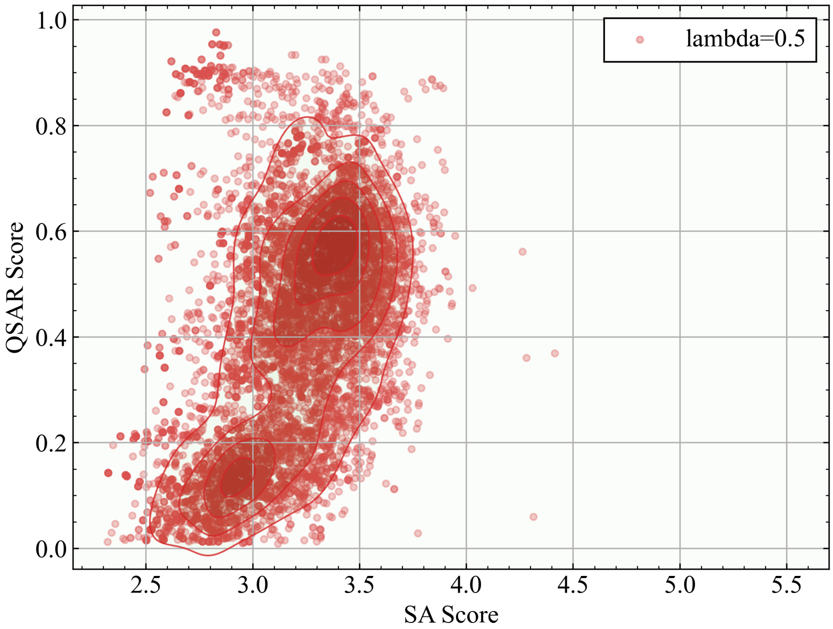

Figure S4: Scatter plot of QSAR values versus SA scores for compounds generated with $\lambda = 0.5$.

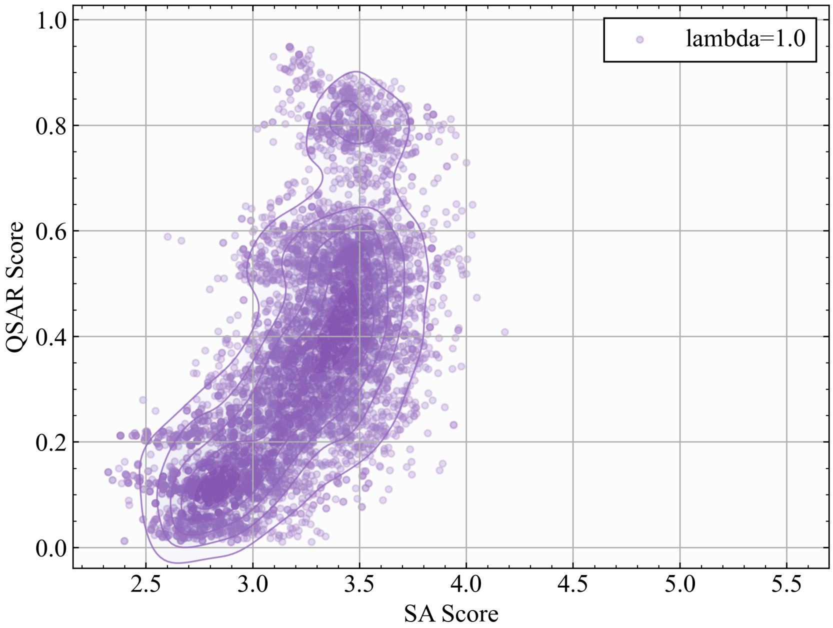

Figure S5: Scatter plot of QSAR values versus SA scores for compounds generated with $\lambda = 1.0$.

References

[1] Samuel Genheden, Amol Thakkar, Veronika Chadimová, Jean-Louis Reymond, Ola Engkvist, and Esben Bjerrum. Aizynthfinder: a fast, robust and flexible open-source software for retrosynthetic planning. *Journal of cheminformatics*, 12(1):70, 2020.

[2] John J Irwin, Khanh G Tang, Jennifer Young, Chinzorig Dandarchuluun, Benjamin R Wong, Munkhzul Khurelbaatar, Yurii S Moroz, John Mayfield, and Roger A Sayle. Zinc20—a free ultralarge-scale chemical database for ligand discovery. *Journal of chemical information and modeling*, 60(12):6065–6073, 2020.

[3] Wenhao Gao and Connor W Coley. The synthesizability of molecules proposed by generative models. *Journal of chemical information and modeling*, 60(12):5714–5723, 2020.
